# Anaerobic bacteria in the intestinal microbiota of Brazilian children

**DOI:** 10.6061/clinics/2017(03)05

**Published:** 2017-03

**Authors:** Silvia T Talarico, Florenza E Santos, Katia Galeão Brandt, Marina B Martinez, Carla R Taddei

**Affiliations:** I Universidade de São Paulo, Faculdade de Ciências Farmacêuticas, Departamento de Análises Clínicas e Toxicológicas, São Paulo/SP, Brazil; II Instituto Médico Professor Fernando Figueira, Recife/PE, Brazil; III Universidade de São Paulo, Escola de Artes, Ciências e Humanidades, São Paulo/SP, Brazil

**Keywords:** Anaerobic Bacteria, Intestinal Microbiota, Brazilian Infants, Real-Time PCR

## Abstract

**OBJECTIVE:**

Changes in the neonatal gut environment allow for the colonization of the mucin layer and lumen by anaerobic bacteria. The aim of the present study was to evaluate *Bifidobacterium*, *Lactobacillus* and *Lactococcus* colonization through the first year of life in a group of 12 Brazilian infants and to correlate these data with the levels of *Escherichia coli*. The presence of anaerobic members of the adult intestinal microbiota, including *Eubacterium limosum* and *Faecalibacterium prausnitzii*, was also evaluated.

**METHODS:**

Fecal samples were collected during the first year of life, and *16S rRNA* from anaerobic and facultative bacteria was detected by real-time PCR.

**RESULTS:**

*Bifidobacterium* was present at the highest levels at all of the studied time points, followed by *E. coli* and *Lactobacillus*. *E. limosum* was rarely detected, and *F. prausnitzii* was detected only in the samples from the latest time points.

**CONCLUSION:**

These results are consistent with reports throughout the world on the community structure of the intestinal microbiota in infants fed a milk diet. Our findings also provide evidence for the influence of the environment on intestinal colonization due to the high abundance of *E. coli*. The presence of important anaerobic genera was observed in Brazilian infants living at a low socioeconomic level, a result that has already been well established for infants living in developed countries.

## INTRODUCTION

Intestinal microbiota play an important role in immunity development 1, nutrition 2 and health 3. The intestinal environment is known to change during the first weeks of a child’s life 4. Soon after birth, the child’s gut has classically been described as initially dominated by a range of facultative bacteria, such as representatives of Enterobacteriaceae, *Streptococcus*, and *Staphylococcus* 5. Once the available oxygen is consumed, strict anaerobes, including species of *Bifidobacterium*, *Bacteroides*, and *Clostridium*, proliferate 6,7. At the end of the first year of life, the intestinal microbiota is mostly composed of anaerobic bacteria.

At the time of weaning, the populations of *Bifidobacterium* and *Lactobacillus* remain highly abundant in the intestinal microbiota, even after the introduction of solid foods to milk-fed infants 9,10. Indeed, some probiotic species from these genera are able to control the composition of the microbiota due to their production of lactate and acetate in the gut environment, and these products play beneficial roles in host health maintenance 11,12.

*Faecalibacterium prausnitzii* belongs to the *C. leptum* group of bacteria (Firmicutes) and is a highly active member of the adult intestinal microbial community that exhibits anti-inflammatory effects 13. Studies of intestinal microbiota based on DNA methodologies have reported a high abundance of *F. prausnitzii* in healthy adults 13, but this species is rarely present in the microbiota of newborns 14.

Our group described the microbial profiles of Brazilian newborns and infants after constructing a *16S* *rRNA* library 8,15. Phylogenetic analysis based on *16S* *rRNA* library construction has been widely used to characterize human fecal microbiota over the last two decades 16-18. However, *16S* *rRNA* library construction may result in a less sensitive assessment of bacterial diversity 19,20, possibly due to bias involved in PCR-dependent methodologies 8.

Indeed, in our previous reports 8,15, we were unable to detect *Bifidobacterium* and some members of adult-like intestinal microbiota, such as *Faecalibacterium prausnitzii* 13 and *Eubacterium limosum* 21, even in older children. *Lactobacillus* was detected with low frequency and abundancy, and *Bifidobacterium* was only detected using qPCR methodology 8,15. qPCR has been widely applied for the quantification of bacterial DNA in different human samples, such as feces 22 and human milk 23, due its specificity and accuracy.

Brazilian newborns exhibited high relative abundances of *Escherichia* and *Clostridium* spp. in neonatal samples, whereas *Staphylococcus* and *Bacteroides* spp. were detected at low frequencies and abundances 15. In infants, the microbial community was composed of aerobic species of *Bifidobacterium* and *Clostridium*, with a high abundance of facultative anaerobe *Escherichia* 15. The pattern of colonization has been noted to differ from that observed for neonates living in developed countries 5,24.

Due to the importance of *Bifidobacterium* and *Lactobacillus* in the intestinal microbiota and because of the bias resulting from the methodology used 8, the aim of the present study was to use qPCR to investigate the process of *Bifidobacterium* and *Lactobacillus* colonization through the first year of life in a group of Brazilian infants living in low socio-economic conditions. We then correlated these data with the levels of *Escherichia coli*. The presence of anaerobic members of the adult-like intestinal microbial community (*Eubacterium limosum* and *F. prausnitzii*) was also evaluated to determine when these species are introduced into the intestinal microbiota of Brazilian children.

## METHODS

### Subjects and Study Design

Twelve infants, selected as previously described 15, were enrolled in this study. Briefly, all infants born by vaginal delivery at the University Hospital were followed monthly throughout the first 12 months of life. During the follow up period, information on breastfeeding, types of food consumed, eventual illnesses, antibiotic treatments, and social or economic disorders were gathered for each child during medical visits with a pediatrician. Fecal samples were collected at the hospital on the 2^nd^ day after delivery (time point 1) and by the mother at home on the 7^th^ (time point 2) and 30^th^ days (time point 3) and at 3, 6 and 12 months of age (time points 4, 5 and 6, respectively). Among the 12 infants enrolled in this study, 8 of the mothers collected fecal samples at the 13^th^, 14^th^ and 15^th^ months of age, and those samples were stored at -80°C. The mothers were instructed to collect the fecal sample with a standardized spoon on the day of the medical appointment, immediately after elimination in a diaper, place it in a sterile plastic container, and store it in a freezer (-20°C) until the appointment a number of hours later. The samples were transported to the laboratory in an ice-filled polystyrene container.

### Study population

All of the infants were exclusively breastfed (100%) for the first 30 days after delivery. During the remaining study period, a portion of the infants were exclusively breastfed until the end of the 5^th^ month, whereas another portion were partially breastfed and supplemented with formula milk without prebiotic components (25% at the end of the 3^rd^ month). All of the infants were eating solid food by the end of the 12^th^ month of age 8. All of the infants lived in a low socioeconomic community and attended a day-care facility. Eight infants received oral antibiotics during the study period. These antibiotic treatments were prescribed for 7 or 10 days to treat respiratory infections, such as bronchiolitis, pneumonia, sinusitis and otitis 8.

### DNA extraction

DNA was extracted from the stool samples using the QIAamp DNA Stool Mini-Kit (Qiagen, Canada) according to the manufacturer’s instructions. The extracted DNA was stored at -20°C until qPCR analysis.

### Real-Time PCR Assays

Real-time PCR (qPCR) was performed on DNA isolated from fecal samples to detect and quantify the presence and abundance of the following anaerobic and facultative anaerobic bacteria: *Bifidobacterium*, *Lactococcus*, *Lactobacillus, E. coli*, *E. limosum* and *F. prausnitzii*. The reactions were performed using a 7500 Fast Real-Time PCR System (Applied Biosystems, Foster City, CA). The *Bifidobacterium animalis* subsp. *lactis* HN019, *Lactococcus lactis* subsp. *lactis* and *Lactobacillus acidophilus* ATCC 4356 strains were cultured in Lactobacilli MRS Broth (Difco) at 37°C under anaerobic conditions, *Escherichia coli* ATCC 25922 were cultured in TSB (Difco) at 37°C under aerobic conditions. *F. prausnitzii* ATCC 27766 were cultured following ATCC^®^ instructions. The genomic DNA from each bacterium was used to generate the standard curves and was extracted with the Wizard Genomic DNA Purification Kit (Promega) following the manufacturer’s instructions. The bacterial genomic DNA was used as a positive control for each corresponding reaction. Genomic DNA from *Eubacterium limosum* ATCC 8486 was obtained directly from the ATCC^®^. To quantify the copy numbers of the *16S rRNA* gene in the fecal samples, standard curves for the relationship between *16S rRNA* gene copy number and threshold cycle (Ct) values were created by analyzing 10-fold serial dilutions of species-specific bacterial strains. Only the results with a reaction efficiency ratio above 95% were considered. DNA of *Bifidobacterium animalis* subsp. *lactis* HN019 and *Escherichia coli* ATCC 25922 were used to construct the standard curves for the total bacteria experiments as described by Furret et al. 25. The results were expressed as log_10_ of the *16S rRNA* copy number/g of feces. Technical triplicates were prepared for the amplification reactions for all of the bacterial strains. The total reaction volume of 20 µL contained 2 µL of DNA extracted from feces, as described earlier, and the remaining volume was composed of master mix (1x), primers and probes (Table 1). The amplification reactions for *Bifidobacterium* spp. and total bacteria were performed in a TaqMan^®^ system using the following program: 50°C for 2 min, 95°C for 10 min, and 40 cycles of 95°C for 15 s and 60°C for 1 min. The amplification reactions for *Lactococcus*, *Lactobacillus*, *E. limosum* and *F. prausnitzii* were performed using the following program on a SYBR^®^ Green I system: 50°C for 2 min, 95°C for 10 min, and 40 cycles of 95°C for 15 s and 60°C for 1 min. A melting step was added to evaluate and optimize the amplification specificity (95°C for 15 s, 60°C for 1 min, 95°C for 15 s and 60°C for 15 s).

### Ethical considerations

This research was approved by the Ethics Committee of the HU-USP (under registration number 574/05). All of the mothers enrolled in the research signed an informed consent form.

## RESULTS

### Inter-individual variation

**Figure 1:**
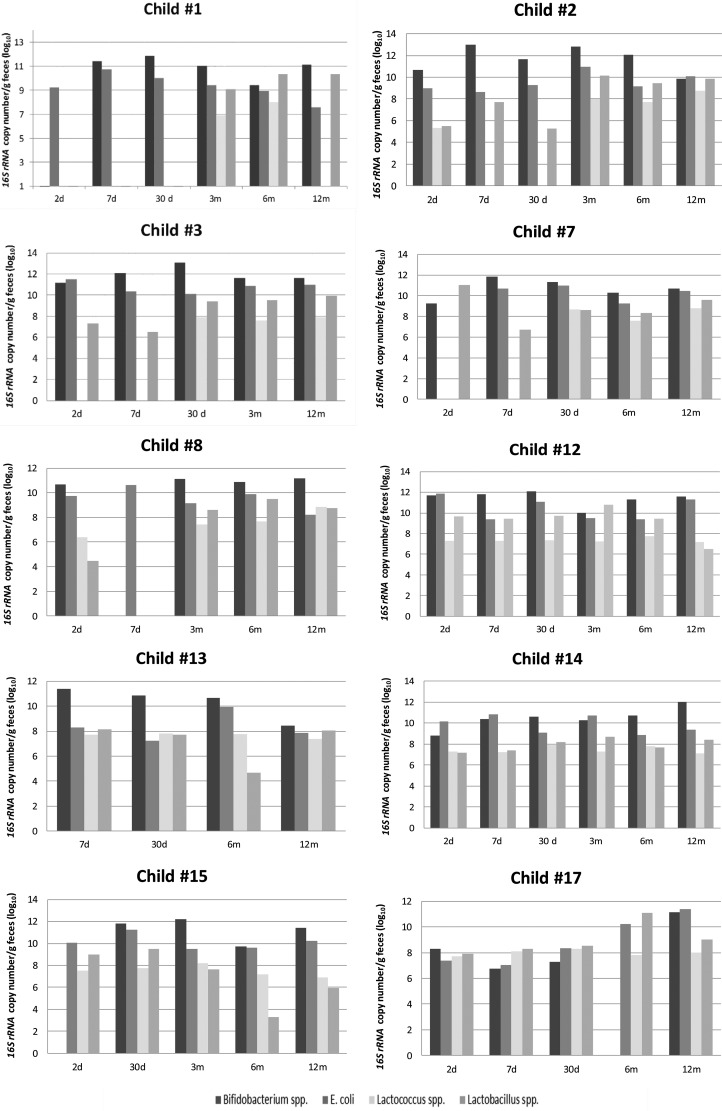
Inter-individual quantification of anaerobic and facultative bacteria in the intestinal microbiota, expressed as log_10_ of *16S rRNA* copy number/g of feces. Time points: 2 days; 7 days; 30 days; 3 months; 6 months; 12 months of age.

The *16S rRNA* copy number of *Bifidobacterium, E. coli, Lactobacillus* and *Lactococcus* was quantified for each child enrolled in this study. Not all requested samples were delivered for children #6 and #16; thus, these children were excluded from the individual variation analysis. The individual results showed a distinct pattern of colonization in the initial days after birth (Figure 1), with a predominance of *Bifidobacterium*.

The bacterial load differed among the children, with variations of 3 log units (Figure 1), and 5 log units for children #7 and #17 in the first days of life. During the following months, the bacterial load of each studied genera increased, with some inter-individual variation.

*Bifidobacterium* was undetectable on the second day for children #1 and #15 and on the seventh day for child #8. A predominance of *E. coli* was observed on the second or seventh day for child #3, child #8, child #12 and child #14. The pediatrician reports for those six children indicated important external factors, such as the usage of antibiotics by mother during pregnancy (#1 and #12) or by the child at the 7^th^ day (#3) or poor sanitary conditions (#8, #14 and #15).

Despite some intra-individual variations in the *16S rRNA* copy number, after the 3^rd^ month, the microbial pattern was similar for all children, with a predominance of *Bifidobacterium* followed by *E. coli* and *Lactobacillus* and the lowest counts of *16S rRNA* copy number for *Lactococcus* until the end of the first year of age. *Bifidobacterium* was undetectable at the sixth month of age for child #17, and the pediatrician records indicated a respiratory infection and antibiotic prescription at that time.

*F. prausnitzii* was detected at some time points (Table 3). The abundance of this species had increased remarkably by the end of the first year of life, when it was detected in 45% of the infants. For the infants from whom fecal samples were collected after 12 months of age, the abundance of the *16S rRNA* gene of *F. prausnitzii* was evaluated to determine its presence and abundance at these later time points. Of the 12 infants enrolled in this study, five of them were positive for *F. prausnitzii* during the first year, with values ranging from 5.7 log_10_ to 15.39 log_10_ copies/g of feces (Table 3). Of the eight infants from who samples were collected after the 13^th^ month of age, 7 had higher detectable levels of *F. prausnitzii* (Table 3). *E. limosum* exhibited the lowest abundances and frequencies among the evaluated anaerobic bacteria. The few samples in which this species was detected were collected from child #8 at time point 1 (4.83 log_10_ copies/g of feces), from child #15 at time point 2 (4.0 log_10_ copies/ g of feces) and from child #6 at time point 4 (7.5 log_10_ copies/g of feces). After 12 months of age, none of the children had detectable *E. limosum*.

### Time point variation

**Figure 2:**
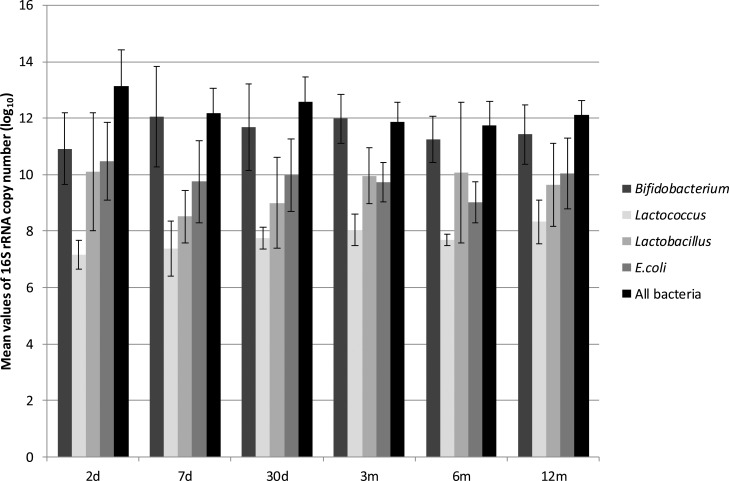
Quantification of anaerobic and facultative bacteria in the intestinal microbiota of infants, expressed as log_10_ of *16S rRNA* copy number/g of feces. Time points: 2 days; 7 days; 30 days; 3 months; 6 months; 12 months of age.

The quantification of the total bacteria at each time point revealed the highest values on the second day of life, with levels that were one or two log units higher than the average values (Figure 2, Table 2). At time point 1 (2 days of age) and throughout the first year, the mean values of total bacteria at each time point did not change significantly, and the differences between time points were on the order of 1 log unit (Figure 2, Table 2).

*Bifidobacterium* was not detected at some time points for a few children, as mentioned previously (Figure 1). Of the anaerobic genera evaluated, *Bifidobacterium* was present in the greatest numbers at all of the time points tested, with maximum values after time point 3 (3 months of age). The mean values of the abundance of *Bifidobacterium* were similar at time points 3, 4 and 5 (Table 2). The abundance of *Bifidobacterium* did not appear to be associated with variations in diet (proportion of breast milk or formula milk).

*E. coli* was found in all of the children and at all time points (Table 2), and the *16S rRNA* copy numbers were the second most abundant among the study subjects. The maximum *16S rRNA* values were detected at time point 1 (Figure 2), and then the mean values ranged from 9.75 log_10_ copies/g of feces on the 7^th^ day to 10.04 log_10_ copies/g of feces at the 12^th^ month, with a minimum value at time point 4 (Table 2). At time points 3 and 4, the mean values for *Lactobacillus* were higher than those for *Escherichia*; however, this was seen in all of the children.

*Lactobacillus* spp. and *Lactococcus* spp. were not detected in some children, (Figure 1, Table 2), with mean abundances that were between 1 and 3 log units lower than the abundances of *Bifidobacterium* (Figure 2). The maximum abundance of *Lactobacillus* was observed at time point 1 (Table 2), and the minimum abundance was observed at time point 2 (Figure 2). The mean abundance values of *Lactococcus* at each time point did not change significantly over the course of the study, and the differences among them were on the order of 1 log unit (Figure 2, Table 2).

## DISCUSSION

The results of quantification of the total bacteria *16S rRNA* copy number in the feces of the children enrolled in this study were similar to those reported in the literature 22,26, and some inter-individual variation was observed throughout the observation period. The data revealed that the bacteria population in the gut was highest at the time the delivery. However, the *Bifidobacterium* counts increased and the *E. coli* counts decreased at the subsequent time point. These data are consistent with reports throughout the world that the intestinal ecosystem shifts toward an anaerobic environment after birth, and the levels of anaerobic bacteria increase 6.

*Bifidobacterium* is an important genus of gut microbiota, and some species play beneficial roles in maintaining host health 12. Breastmilk is both an important source of *Bifidobacterium* for infants 29 and also a source of carbohydrates, which promote *Bifidobacterium* colonization, even in mixed feeding diets 10. The infants enrolled in this study were breastfed during their early life, and even during the introduction of formula milk, the mother’s milk was also present in their diet, characterizing the diet as mixed feeding 8. *Bifidobacterium* was not detected at some time points for a few children. Interestingly, the pediatrician reports indicated the use of antibiotics by some of these children before sample collection or the prescription of antibiotic treatment to the mothers for urinary tract infection during the last trimester of pregnancy. The ability of antibiotic treatment to disturb the microbiota composition, including a decrease in *Bifidobacterium* abundance, has been reported previously 10. Although our sample size is limited, our results suggested that such changes may also occur in children.

However, *Bifidobacterium* was the predominant species detected in this group of infants at twelve months of age, corroborating the known benefits of breastfeeding as a source of *Bifidobacterium* and its maintenance in the gut mucosa 9,10. This genus has been described throughout the world as the predominant bacterial group detected in the feces of infants 26-28. Our data showed a lower *16S rRNA* copy number of *Bifidobacterium* after six months of age, potentially because of the introduction of new genera of bacteria via solid foods 30.

*Lactobacillus* colonization at birth has been attributed to the maternal vaginal flora 31 and, possibly, to the presence of *Lactobacillus* in the womb environment 32. These findings may explain the observation that the maximum abundance of *Lactobacillus* occurred on the second day of life in Brazilian infants, followed by a decrease at the seventh day and an increase after one month of age. These data suggest that known environmental changes 6 cause a decrease in the initial levels of the maternal microbiota and that an infant’s microbiota has begun to become established by this time; the increase in the abundance of *Lactobacillus* after the first month of life highlights the fact that breast milk is an important natural promoter of this bacterial genus.

The inter-individual analysis showed that, in the first days of life, microbiota colonization is affected by individual exposure to environmental factors; in subsequent months, the pattern of anaerobic and facultative genera colonization appears to be mediated by the milk diet, with a predominance of *Bifidobacterium* and, with lower abundance, *Lactobacillus*, supporting global knowledge about the role of dietary milk in infant intestinal colonization 9,10.

Because our previous study detected *Escherichia* in high abundance in this group of children based on library construction 8, we quantified *Escherichia* at different time points. At some time points, particularly at the second and 12^th^ months, the copy numbers of *Escherichia 16S rRNA* were the second highest, consistent with our previous results 8,15. However, due to the presence of *Bifidobacterium* and *Lactobacillus* and their protective properties 11 in these childreńs feces, lower values of *Escherichia* than those observed in the present study were expected. In a study of healthy children in Africa 33, a high proportion of *Escherichia* was detected in the intestinal microbiota in children ranging between zero and 11 months of age. The environmental forces controlling the establishment of the fecal microbiota may favor the maintenance of *E. coli* in this community 8,15, as well as in other developing countries 33.

*F. prausnitzii* is a well-established member of the adult intestinal microbiota with anti-inflammatory properties 13. A few papers have described the presence of this species in children’s feces in other populations 28,33,34, but this adult-like bacterium has not been detected in Brazilian infants’ feces. In the present study, this bacterial species was only observed in the fecal microbiota of two newborns on the second and seventh days of life, suggesting maternal transmission. These results suggest that the intestinal environment is unfavorable for its maintenance. After the sixth month of life, a gradual increase in the colonization quantity and frequency was observed. Interestingly, the majority of the fecal samples collected from children after one year (at 13-15 months of age) had greater abundances of *F. prausnitzii*. These findings are in accordance with those published by Hopkins et al. 28 and Pop et al. 33, in which the abundance of this species increased at the end of the first year of life. These data suggest that until the sixth month of life, the intestinal environment is unfavorable for the establishment of *F. prausnitzii* and that with the introduction of solid food and the development of a more stable environment, this genus becomes an important intestinal colonizer. Lin et al. 34 reported the presence of *F. prausnitzii* in older children from both the USA and Bangladesh, with a higher abundance in American children. However, the role of environmental factors in the abundance of *F. prausnitzii* was not discussed, and more studies are needed to determine whether there is any relationship with external factors.

*E. limosum* is an anaerobic Gram-positive rod present in the colon of adult humans. This species has a butyrate-producing capacity and consequently has beneficial effects in inflammatory bowel disease 35. In this study, *E. limosum* was detected in four infants at different time points, indicating its presence in the environment and, therefore, demonstrating that exposure to this genus occurs early in life, although members of this genus have a low capacity for colonization of the intestinal milieu until the 12^th^ month of life.

The present study reports the characterization of the fecal microbiota in Brazilian infants, which is dominated by *Bifidobacterium* and *Lactobacillus*. The absence of *Eubacterium limosum* and the late colonization of *F. prausnitzii* are also notable. These findings suggest a lack of adult-like microbiota in infants, corroborating the results of a previous study by Ringel-Kuka et al. 36. These results contribute to observations throughout the world of the establishment of the intestinal microbiota of infants fed milk diets. The high abundance of *E. coli* suggests a pattern related to unhygienic conditions, as reported previously in developing countries 34. These results complement analyses of the composition of the gut microbiota in this group of Brazilian breastfed infants living in low socio-economic conditions 8,15 and highlight the influence of both diet and the environment.

## AUTHOR CONTRIBUTIONS

Talarico ST performed the experiments and participated in data analysis. Santos FE performed some experiments. Brandt KG selected the children, followed the medical appointments and collected the samples. Martinez MB designed the study and participated in manuscript writing. Taddei CR designed the study, followed the experiments, conducted the data analysis and wrote the manuscript.

## Figures and Tables

**Table 1:** Primers and probes used in this study.

Target	Primers and probes	Sequence (5ʹ – 3ʹ)	Conc. (nM)	Reference
*Bifidobacterium* spp.	F_Bifid 09c	CGG GTG AGT AAT GCG TGA CC	300	25
	R_Bifid 06	TGA TAG GAC GCG ACC CCA	300	
	P_Bifid	6FAM-CTC CTG GAA ACG GGT G	250	
*Eubacterium limosum*	LIMO-F	TGG ATC CTT CGG GTG ACA TT	300	21
	LIMO-R	CTC ATT GGG TAC CGT CAT TC	300	
*Lactobacillus* spp.	Lactobacillus F	AGC AGT AGG GAA TCT TCC A	300	19
	Lactobacillus R	CAC CGC TAC ACA TGG AG	300	
*Lactococcus* spp.	Llac05-F	AGC AGT AGG GAA TCT TCG GCA	300	25
	Llac02-R	GGG TAG TTA CCG TCA CTT GAT GAG	900	
*Faecalibacterium prausnitzii*	Fprau 07	CCA TGA ATT GCC TTC AAA ACT GTT	300	13
	Fprau 02	GAG CCT CAG CGT CAG TTG GT	300	
All bacteria	F_Bact 1369	CGG TGA ATA CGT TCC CGG	200	25
	R_Prok 1492	TAC GGC TAC CTT GTT ACG ACT T	200	
	P_TM 1389F	6FAM-CTT GTA CAC ACC GCC CGT C	250	

**Table 2:** Minimum, maximum and mean *16S rRNA* copy number of bacteria in feces of infants. Values expressed as log_10_.

	*Bifidobacterium*			*Lactobacillus*			*Lactococcus*			*Escherichia coli*			*All bacteria*		
	min. values	max values	average (SD)	min. values	max values	average (SD)	min. values	max values	average (SD)	min. values	max values	average (SD)	min. values	max values	average (SD)
2 d	ND	11.18	10.9 (±1.27)	ND	11.04	10.10 (±2.1)	ND	7.74	7.16 (±0.52)	8.53	11.14	10.48 (±1.37)	9.76	14.12	13.13 (±1.28)
7 d	ND	12.98	12.06 (±1.78)	ND	9.45	8.51 (±0.92)	ND	8.08	7.38 (±0.97)	6.33	10.16	9.75 (±1.46)	10.37	12.56	12.19 (±0.88)
1 mo	10.09	12.08	11.68 (±1.54)	ND	9.74	9.00 (±1.6)	ND	7.95	7.75 (±0.39)	6.51	10.52	9.98 (±1.28)	10.76	13.25	12.57 (±0.88)
3 mo	10.00	12.81	11.98 (±0.87)	ND	10.79	9.96 (±0.99)	7.22	8.67	8.03 (±0.56)	8.45	10.28	9.72 (±0.70)	10.53	12.38	11.85 (±0.72)
6 mo	ND	12.06	11.25 (±0.82)	ND	8.03	10.08 (±2.49)	ND	12.06	7.69 (±0.20)	8.18	10.18	9.02 (±0.72)	9.87	12.54	11.74 (±0.85)
12 mo	8.43	12.03	11.42 (±1.05)	5.95	10.34	9.64 (±1.47)	ND	8.83	8.33 (±0.77)	7.53	10.69	10.04 (±1.26)	11.45	12.55	12.10 (±0.51)

ND – not detected

**Table 3:** Quantification of *Faecalibacterium prausnitzii* in feces of infants.

Child	Age	copy number (log_10_)
1	12 months	ND**
	13 months	7.59
2	12 months	7.24
	13 months	10.92
3	12 months	9.24
	13 months	NA*
6	12 months	ND**
	13 months	10.29
7	12 months	ND**
	13 months	ND**
	14 months	6.31
	15 months	7.64
8	12 months	9.73
	13 months	NA*
12	2 days	5.70
	12 months	8.44
	13 months	9.66
	14 months	ND**
13	12 months	ND**
	16 months	9.90
14	7 days	6.77
	12 months	6.56
	13 months	NA*
15	4 months	7.54
	6 months	6.44
	12 months	ND**
	15 months	ND**
16	12 months	NA*
17	12 months	ND**
	15 months	7.08

* NA – not available

** ND – not detected
